# Hydroxytyrosol Protects against Oxidative DNA Damage in Human Breast Cells

**DOI:** 10.3390/nu3100839

**Published:** 2011-10-13

**Authors:** Fernando Warleta, Cristina Sánchez Quesada, María Campos, Yosra Allouche, Gabriel Beltrán, José J. Gaforio

**Affiliations:** 1 Immunology Division, Department of Health Sciences, Faculty of Experimental Sciences, University of Jaén, Campus las Lagunillas s/n, 23071 Jaén, Spain; Email: fwarleta@ujaen.es (F.W.); csquesad@ujaen.es (C.S.Q.); mcampos@ujaen.es (M.C.); yosraallouche@yahoo.fr (Y.A.); 2 Agrifood Campus of International Excellence, ceiA3, Spain; 3 Instituto Andaluz de Investigación y Formación Agraria, Pesquera y de la Producción Ecológica (IFAPA), Centro “Venta del Llano”, 23620 Mengibar, Spain; Email: gabriel.beltran@juntadeandalucia.es

**Keywords:** breast cancer, Mediterranean diet, olive oil minor compounds, hydroxytyrosol, tyrosol, phenols, oxidative stress, reactive oxygen species, DNA damage

## Abstract

Over recent years, several studies have related olive oil ingestion to a low incidence of several diseases, including breast cancer. Hydroxytyrosol and tyrosol are two of the major phenols present in virgin olive oils. Despite the fact that they have been linked to cancer prevention, there is no evidence that clarifies their effect in human breast tumor and non-tumor cells. In the present work, we present hydroxytyrosol and tyrosol’s effects in human breast cell lines. Our results show that hydroxytyrosol acts as a more efficient free radical scavenger than tyrosol, but both fail to affect cell proliferation rates, cell cycle profile or cell apoptosis in human mammary epithelial cells (MCF10A) or breast cancer cells (MDA-MB-231 and MCF7). We found that hydroxytyrosol decreases the intracellular reactive oxygen species (ROS) level in MCF10A cells but not in MCF7 or MDA-MB-231 cells while very high amounts of tyrosol is needed to decrease the ROS level in MCF10A cells. Interestingly, hydroxytyrosol prevents oxidative DNA damage in the three breast cell lines. Therefore, our data suggest that simple phenol hydroxytyrosol could contribute to a lower incidence of breast cancer in populations that consume virgin olive oil due to its antioxidant activity and its protection against oxidative DNA damage in mammary cells.

## 1. Introduction

Olive oil is the major source of fats in the Mediterranean diet and is considered to be responsible for the health benefits associated with this diet. In fact, it has been demonstrated that people who consume virgin olive oil (VOO) present a lower incidence of several cancers, including breast cancer [1]. This effect has previously been attributed to the high content of monounsaturated fatty acids. However, more recently, the importance of the minor constituents of olive oil has been considered [2]. Over the last five decades, several publications have firmly established that ingestion of small quantities of certain compounds isolated from plants can lower the risk of cancer in mammals exposed to carcinogens, including polyphenols [3].

VOO contains relatively high amounts of minor compounds compared to other oils (refined olive oil or seed oils). Among these, phenolic compounds are present at levels between 200 and 1500 mg/kg [4] depending on the olive tree variety, climatic and agronomic conditions, degree of maturation at harvest, and the manufacturing process [4]. At present, there are many studies reporting biological activities *in vitro*, *in vivo* and in clinical assays of phenolic compounds naturally present in VOO. Between them, anti-inflammatory, cardioprotective antioxidant and chemopreventive effects in breast and other types of cancers have been defined [5]. The major phenols identified in olive oils include the simple phenols hydroxytyrosol (HT) and tyrosol (TY), secoiridoids and lignans [2]. The concentration of TY is always higher than of HT [6]. Hydrolysis of secoiridoid during olive oil storage results in the formation of HT and TY [7].

It has been well established that HT is a potent antioxidant because of its marked antioxidant activity, its ability to scavenge oxygen and nitrogen free radicals, to inhibit Low Density Lipoprotein (LDL) oxidation, platelet aggregation and endothelial cell activation and its protection against DNA damage [2,8]. HT was able to reduce the synthesis of prostaglandin E2 blocking the transcription of COX-2 and 5-lipooxygenase, thereby reducing the chronic influence associated with diseases such as cancer [9]. TY has been described as exerting a weak antioxidant activity, although it is able to scavenge peroxynitrite and superoxide radicals, inhibit LDL oxidation in Caco2 cells and inhibit LPS-induced cytokines release from human monocytes [10,11].

It has been suggested that HT and TY compounds might have preventive activity against breast cancer, but, at present, the exact role played by these phenols in breast cancer prevention is still unknown. In this sense, despite epidemiological evidence, *in vitro* experiments have not been conducted to check if there are different effects of the simple phenols HT and TY between human breast cancer cells and human breast non-cancer cells.

The present study attempts to provide new insights into the antioxidant capacity of HT and TY and the *in vitro* effects on proliferation, cell cycle progression, apoptosis, reactive oxygen species (ROS) production and oxidative DNA damage in the human breast epithelial MCF10A cell line and the human breast MCF7 and MDA-MB-231 cancer cell lines.

## 2. Experimental Section

### 2.1. Chemicals and Materials

The following were purchased from Sigma-Aldrich Co. (St Louis, MO, USA): Hepes Buffer; Sodium Pyruvate; Non-Essential Amino Acids mixture 100× (NEAA); 2′,7′-dichlorofluorescein diacetate (DCFH-DA); Dimethyl sulfoxide (DMSO); 2,3-Bis(2-methoxy-4-nitro-5-sulfophenyl)-2*H*-tetrazolium-5-carboxanilide inner salt (XTT sodium salt) purity ≥90%; *N*-Methylphenazonium methyl sulfate (PMS) purity ~98%; 2-hydroxyphenyl ethanol (Tyrosol, CAS 501-94-0 (TY)) purity 98%; DL-all-rac-α-Tocopherol (Vitamin E, CAS 10191-41-0 (TOC)) purity ≥96%; 6-Hydroxy-2,5,7,8-tetramethylchroman-2-carboxylic acid (Trolox™ CAS 53188-07-1 (TR)) purity ≥97%; 2,2′-Azobis (2-methylpropionamidine) dihydrochloride (AAPH) purity ~97%; 2,2-Diphenyl-1-picrylhydrazyl (DPPH) purity ~90%, (*S*)-(+)-camptothecin (CAS 7689-03-4 (CPT)) purity ~95%; 2,2′-azino-bis(3-ethylbenzthiazoline-6-sulphonic acid) diammonium salt tablets (CAS 30931-67-0 (ABTS)); PBS; HBSS. 2-(3,4-dihydroxyphenyl) ethanol (Hydroxytyrosol, CAS 10597-60-1 (HY)) purity ≥98% was obtained from Cayman Chemical (Ann Arbor, MI, USA). Minimum essential medium with Eagle’s salts (MEM), Fetal Bovine Serum (FBS) and Phenol-Red-free Roswell Park Memorial Institute 1640 medium (RPMI) were obtained from PAA Laboratories GmbH (Pasching, Austria). TrypLE Express, HuMEC Ready Medium kit and Fluorescein (FL) were obtained from Invitrogen (Eugene, OR, USA). K_2_S_2_O_8_ (CAS 7727-21-1) was obtained from Panreac Quimica S.A.U. (Barcelona, Spain). Culture plates were obtained from NUNC™ (Roskilde, Denmark). The PI/RNase Staining Buffer kit, FITC-conjugated Annexin V and Binding Buffer were obtained from BD Biosciences Pharmigen (San Diego, CA, USA). The Comet assay kit was obtained from Trevigen, Inc. (Helgerman CT, Gaithersburg, MD, USA). 

### 2.2. DPPH Assay

The antioxidant activity of HT and TY against the stable radical DPPH was measured as previously reported [12] with some modifications. Briefly, 100 µM ethanolic solution of DPPH was mixed with different ethanolic solutions of HT or TY in 96-well plates at 0.06, 0.13, 0.25, 0.5 and 1 (moles of antioxidant/moles of DPPH). (±)-α-tocopherol (TOC) was used as a positive control and a sample without antioxidant was also measured as a blank control. The decrease in absorbance at 520 nm was determined immediately and every 5 min for 2 h in a microplate reader (TECAN, GENios Plus). Measurements were performed in triplicate.

The inhibition of the DPPH radical was calculated according to the following percentage of Radical Scavenging Activity (% RSA) formula:

% RSA = [(*A*_C(0)_ − *A*_A(*t*)_)/*A*_C(0)_] × 100

where *A*_C(0)_ is the absorbance of the control at *t* = 0 min and *A*_A(*t*)_ is the absorbance of the antioxidant at *t* = 50 min.

### 2.3. ABTS Assay

ABTS cation radical scavenging activity was determined using a previously reported procedure [13]. ABTS radicals (ABTS^•+^) were obtained by ABTS/H_2_O 0.5 mM reaction with K_2_S_2_O_8_ for 16 h in the dark at room temperature. ABTS^•+^ was diluted in ultrapure water until absorbance at 734 nm was 0.7 (±0.1). HT, TY and Trolox™ (TR) (as antioxidant reference) was dissolved in ethanol to yield a 10 mM stock solution and diluted with ultrapure water to the assayed concentrations. Twenty microliters of each concentration of HT, TY, standard (TR), blank (ultrapure water) or ethanol control (8%) were added to a 96-well plate. The reaction was initiated by the addition of 280 µL of ABTS^•+^. Absorbance readings were taken every 5 min at 30 °C for 2 h in a microplate reader (TECAN, GENios Plus). All determinations were carried out in triplicate. 

The inhibition of ABTS^•+^ was calculated according to the percentage of Radical Scavenging Activity (% RSA) described above (at *t* = 30 min).

### 2.4. ORAC Assay

Peroxyl radical scavenging activity was measured by the ORAC_FL_ assay as previously described [14]. A stock solution of HT or TY were reconstituted in DMSO and then diluted in PBS. A stock solution of TR, as antioxidant standard, was also diluted in DMSO and diluted in PBS. The assay was carried out in 96-well plates with a final volume of 160 μL. Samples were run in triplicate. Fluorescein (48 nM) was mixed with various concentrations of SQ, standard (TR) or blank (PBS) containing at final volume 1% (v/v) DMSO. Plates were incubated for 15 min at 37 °C. The assay was initiated by the addition of AAPH (100 mM) and fluorescence readings (Ex: λ_485_/Em: λ_520_ nm) were taken every 5 min at 37 °C for 160 min in a microplate reader (TECAN GENios Plus). Final results were calculated based on the difference in the Area Under the fluorescence decay Curve (AUC) between the blank and each sample. The AUC formula was:

AUC = 1 + *f*_1_/*f*_0_ + *f*_2_/*f*_0_ + *f*_3_/*f*_0_ + *f*_4_/*f*_0_ + … + *f*_20_/*f*_0_

Results were expressed as micromolar TR equivalents (TE) calculated using the line equation from the standard curve:

TE = (*Y* − *b*)/*m*

where *Y *is the net AUC (AUC_sample_ − AUC_control_), *m* is the slope and *b* is the *Y*-intercept.

### 2.5. Cell Culture

Highly invasive MDA-MB-231 human breast cancer cells (estrogen and progesterone receptor-negative), minimally invasive MCF7 human breast cancer cells (estrogen and progesterone receptor-positive) and immortalized non-tumorigenic MCF10A human breast epithelial cells, were obtained from American Type Culture Collection (ATCC, Rockville, MD, USA). Breast tumor cells were grown as a monolayer culture in Minimum Essential Medium with Eagle’s salts (MEM) supplemented with 10% Fetal Bovine Serum (FBS), 1% Hepes Buffer 1 M, 1% Sodium Pyruvate 100 mM and 1% Non-Essential Amino Acids mixture 100×. MCF10A cells were cultivated in HuMEC Ready Medium. All cell lines were maintained at 37 °C in a humidified incubator with 5% CO_2_. Cells were routinely sub-cultured using TrypLE Express solution. Cells in the exponential growth phase were used for all experiments.

### 2.6. Cell Proliferation Assay

Cell proliferation, measured as the cellular growth of treated cells *vs.* untreated controls, was measured using an XTT-based assay as described by Scudiero *et al.* [15] with some modifications. Briefly, cells were seeded at 2 × 10^3^ cells/well (MCF7) or 1 × 10^3^ cells/well (MDA-MB-231 and MCF10A) into 96-well culture plates (flat bottom) (100 μL of cell suspension/well). At 24 h after plating, 100 μL of fresh culture medium, with different concentrations of HT or TY was added in triplicate to the wells. Plates were incubated for 24 h or 24 h followed by a 48 h proliferation period with fresh medium at 37 °C and 5% CO_2_. At these time points, medium was removed and 200 μL of fresh RPMI medium without phenol red that contained XTT (200 μg/mL) and PMS (20 μg/mL) was added. Plates were incubated for 3 h at 37 °C in 5% CO_2_ and absorbance was measured at 450 nm wavelength (620 nm as reference) in a plate reader (TECAN GENios Plus). Viability was calculated using the formula: 

viable cells (%) = (OD_treated cells_/OD_control_) × 100

where OD is the difference in absorbance between optical density units (OD = OD_450_ − OD_620_).

All measurements were performed in triplicate and each experiment was repeated at least three times.

### 2.7. Cell Cycle Assay

Cells were seeded in 12-well culture plates at 1 × 10^5^ cells/well for MCF7 and MDA-MB-231 or at 5 × 10^4^ cells/well for MCF10A for 48 h. Cells were then treated with different doses of HT or TY for 24 h. After incubation, cells were washed in cold PBS, fixed with cold 70% ethanol and stored at −20 °C for at least 24 h. At least 1 × 10^4^ cells per sample were analyzed on an EPICS XL-MCL (Beckman Coulter, Spain) flow cytometer after propidium iodide labeling (PI/RNase Staining Buffer kit). The percentage of cells in G_0_/G_1_, S and G_2_/M phases were calculated using FlowJo program (v5.7.2). Each experiment was repeated three independent times.

### 2.8. Apoptosis

The percentage of apoptosis was determined using a double staining assay with FITC-conjugated Annexin V and propidium iodide (PI). Briefly, after 24 h of cell exposure to the previously indicated doses of HT or TY in 12-well culture plates, cells were harvested, washed twice in cold PBS and resuspended in 100 μL 1× Annexin Binding Buffer. Cells were then stained with 5 μL Annexin V-FITC and 1 μL PI solution, gently vortexed, and incubated for 15 min at room temperature in the dark before flow cytometric analysis. As a positive control, cells were treated with 1 μM camptothecin (CPT). Each experiment was repeated three independent times.

### 2.9. Reactive Oxygen Species Detection

Intracellular reactive oxygen species (ROS) level was measured using a cell-permeable fluorescent probe, 2′,7′-dichlorofluorescein diacetate (DCFH-DA) as we described previously [16]. In brief, cells were seeded into 96-well culture plates at 1 × 10^4^ cells/well (MCF7, MDA-MB-231 cells) or 5.5 × 10^3^ cells/well (MCF-10A cells). After 24 h at 37 °C and 5% CO_2_, cells were treated with different doses of HT or TY for 24 h. Cells were then washed twice with Hank’s Buffered Salt Solution (HBSS) and incubated with fresh DCFH-DA (100 μM) in HBSS for 30 min at 37 °C in 5% CO_2_. DCFH-DA stock solution (20.5 mM) was prepared in DMSO and stored at −20 °C for maximum one month. After that, cells were washed twice in HBSS, and wells were filled with 100 µL HBSS before fluorescence acquisition in a plate reader (TECAN GENios Plus) (Ex: λ_485_/Em: λ_535_ nm, Gain 60). Intracellular ROS level percentage was calculated as follows: 

F = [(F*_t_*_30_ − F*_t_*_0_)/F*_t_*_0_] × 100

where F*_t_*_0_ is the fluorescence at *t* = 0 min and F*_t_*_30_ the fluorescence at *t* = 30 min.

It has been reported that the addition of H_2_O_2_ increases oxidative stress in cultured cells [17]. Therefore, in order to evaluate the protective capacity of HT or TY against induced oxidative stress, H_2_O_2_ (500 μM) was added to the wells after removal of assay medium. This allows avoiding a direct reaction in the medium between these compounds and the oxidant source. After 30 min at 37 °C, fluorescence was quantified as described above.

All tests were run in triplicate for each experimental condition and each experiment was repeated at least three times. All experiments were conducted using iron-free media (MEM and HuMEC).

### 2.10. Alkaline Single-Cell Gel Electrophoresis (Comet Assay)

At 24 h, cells treated with HT or TY were scraped into 12-well culture plates, washed twice (300× *g* 10 min, 4 °C) with cold 1× PBS (Ca^2+^/Mg^2+^ free) and resuspended in 1 mL of cold 1× PBS. In order to evaluate the ability of HT and TY to prevent oxidative DNA damage, cell suspensions were exposed for 10 min to 50 µM H_2_O_2_ at 4 °C. After that, cells were washed twice and frozen in FBS-DMSO (90:10, v/v) at −80 °C until the Comet assay procedure.

DNA single strand break by alkaline microgel electrophoresis was performed according to Singh *et al*. [18] with some modifications. Cells were thawed in a bath at 37 °C, centrifuged (300× *g* 10 min, 4 °C) in cold MEM with 25% FBS and resuspended in cold 1× PBS to a density of 1.65 × 10^5^ cells/mL. Cells were then suspended in melted and cooled (at 40 °C) low melting point agarose (LMA). Cell suspensions (50 µL) were spread over a sample area of pre-warmed 1% normal melting point agarose (NMA) precoated CometSlide™ slides. After 15 min at 4 °C in the dark, slides were immersed in cold Lysis Solution (Trevigen, Inc.) at 4 °C for 30 min to dissolve lipids and proteins. In order to separate the two DNA strands, slides were then immersed in fresh Alkaline Solution (pH > 13) for 30 min at room temperature in the dark. Electrophoresis was performed in an Ebony acrylic electrophoresis tank with a cooled platform containing cold Alkaline Electrophoresis Solution (300 mM NaOH, 1 mM EDTA, pH > 13) at 25V (1 V/cm) and 300 mA for 40 min. The slides were washed twice with distilled water for 10 min and neutralized with 10 mM Tris-HCl, pH 7.5 for 5 min, followed by immersion in 70% ethanol for 5 min and air-dried overnight at room temperature. Slides were stained with Sybr^®^ green before scoring.

### 2.11. Slide Scoring and Analysis

DNA strand breaks were examined using a fluorescence microscope (Zeiss Axiovert 200) equipped with a Luca EMCCD camera (Andor Technology, Belfast, UK) under 494 nm excitation and 521 nm emission wavelength using the Komet 5.5 software package (Kinetic Imaging Ltd., Liverpool, UK). Fifty cell images were randomly characterized per sample using 20× magnification. Relative fluorescence between head and tail through the olive tail moment (Olive_TM) was used to determine DNA damage. Olive_TM is defined as the product of the Tail Moment Length and the fraction of DNA in the tail. 

Olive_TM = [(Tail (mean) − Head (mean)) × Tail (% DNA)]/100

### 2.12. Statistical Analysis

Results are presented as mean (±SEM), except for cell proliferation results. For this assay, results are presented as mean (±SD). Results are expressed as a percentage relative to the control, which was defined as 100%. Statistical analysis was performed using one-way analysis of variance (ANOVA) followed by Fisher’s least significant difference (LSD) test. Values of *p *< 0.05 were considered significant. Statgraphics Plus 5.1 statistical software (Statpoint Technologies, Inc., Warrenton, VA, USA) was used for the statistical analysis.

## 3. Results

### 3.1. Effect of HT and TY on Radical Scavenging Activity

The antiradical activity of HT and TY, measured by scavenging activity in the DPPH radical assay, indicated that HT at up to 10 mole ratio (mole antioxidant/mole DPPH) exerts a slightly higher scavenging activity than TOC while TY does not possess a radical scavenger activity (Table 1(a)).

The ABTS antiradical assay showed that HT was more effective than TR in scavenging the ABTS cationic radical while TY exhibited a maximum 85% RSA at 800 µM (Table 1(b)).

The peroxyl radical scavenging activity of HT and TY, measured by the ORAC_FL_ assay, showed a protective effect against AAPH-induced peroxyl radical activity for both phenols. Both exerted higher protection against the peroxyl radical than TR for low concentrations up to 100 µM (Table 1(c)).

### 3.2. Cell Proliferation

**Figure 1:**
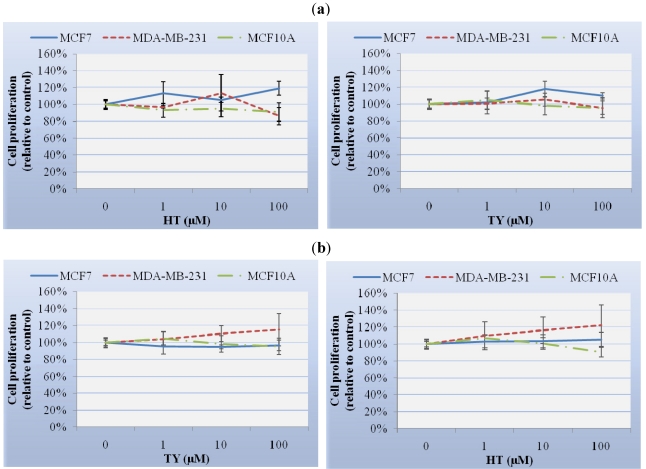
Cell proliferation assay measured with XTT tetrazolium salt (**a**) after 24 h of HT or TY exposure, or (**b**) after 24 h of HT or TY exposure followed by 48 h with fresh medium. Data are the mean (±SD) relative to an untreated control of three independent assays carried out in triplicate.

To investigate the effect of HT and TY on human breast cell growth, cells were treated with concentrations of HT or TY ranging from 1 to 100 μM for 24 h. Neither HT nor TY had significant effects on the cell proliferation rates of MCF7, MDA-MB-231 and MCF10A cells (Figure 1(a)), even after an additional 48 h with fresh medium (Figure 1(b)). We also investigated the potential antiproliferative effect of these compounds at high, non-physiological concentrations up to 1000 or 5000 μM of HT or TY, respectively. HT showed a dose-dependent reduction of cell proliferation in the three cell lines from a concentration of 200 μM with an absence of viability observed at 1000 μM, while TY did not affect cell viability at any concentration assayed (data not shown).

No marked changes in cell morphology were observed by light microscopy in any of the cell lines tested when concentrations between 1 and 100 μM of HT or TY were used (data not shown).

**Table 1:** Antioxidant activity of hydroxytyrosol (HT) or tyrosol (TY) quantified as Radical Scavenging Activity (RSA) by (**a**) DPPH assay (% RSA at 50 min) and (**b**) ABTS assay (% RSA at 30 min); (**c**) Antioxidant activity quantified as Trolox Equivalent (TE) by ORAC_FL_ assay. Trolox™ (TR) and α-tocopherol (TOC) were used as antioxidant references.

(a)			
mole AH/ mole DPPH	HT	TY	TOC
0	3.96	3.64	3.63
0.06	36.24	n.d.	23.21
0.13	71.47	5.20	48.16
0.25	96.40	4.25	75.85
0.5	97.80	3.02	90.17
1	98.07	2.59	95.98
2.5	n.d.	2.34	n.d.
5	n.d.	3.44	n.d.
10	n.d.	3.37	n.d.
n.d.: not determined.			

(b)			
µM	HT	TY	TR
**6**	5.07	10.31	n.d.
**12.5**	5.45	14.32	n.d.
**25**	8.42	20.14	n.d.
**50**	18.09	31.81	16.21
**100**	36.63	44.55	29.39
**200**	69.53	58.25	50.07
**400**	96.30	73.82	88.03
**800**	99.47	85.04	99.48
n.d.: not determined.			

(c)		
TE (µM)	HT (µM)	TY (µM)
**3.12**	14.82	1.64
**6.25**	30.97	8.30
**12.5**	50.37	20.84
**25**	92.24	58.35
**50**	150.43	106.76
**100**	262.76	205.57

### 3.3. Cell Cycle and Apoptosis

To evaluate whether HT or TY interfered with the cell cycle or the induction of apoptosis, MCF7, MDA-MB-231 and MCF10A cells were treated for 24 h with increasing concentrations of HT or TY (between 10 and 200 μM). The results revealed that HT and TY did not alter the cell cycle in any of the cell lines studied (data not shown).

Flow cytometric analysis of apoptosis revealed that treatment with HT or TY for 24 h did not induce apoptosis in MCF10A cells or in MCF7 or MDA-MB-231 cells when compared to the controls (data not shown).

### 3.4. Intracellular ROS Level

**Figure 2:**
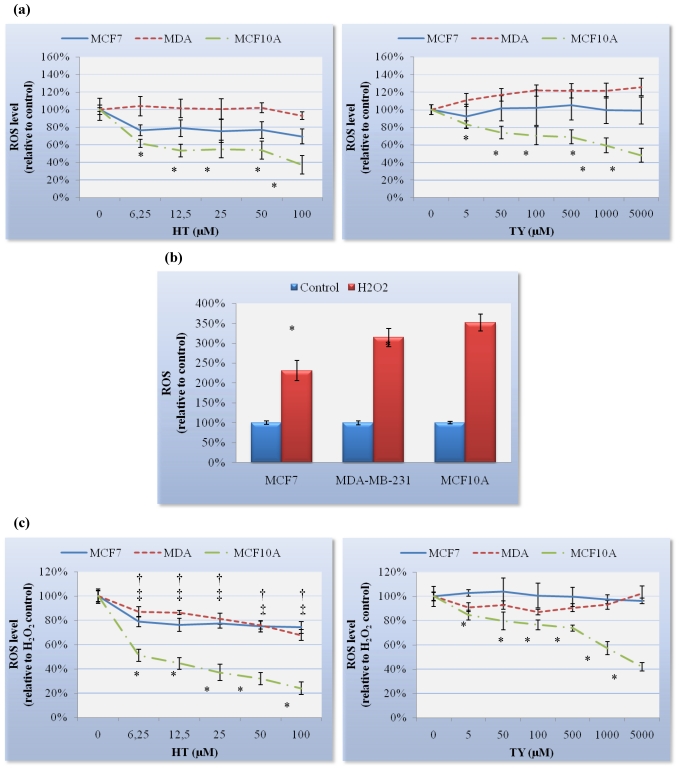
(**a**) Intracellular reactive oxygen species (ROS) in breast cells treated for 24 h with HT or TY; (**b**) Increase of the cellular ROS level after an oxidative burn with H_2_O_2_; (**c**) Intracellular ROS in breast cells treated for 24 h with HT or TY followed by an oxidative burst with H_2_O_2_. Inhibitory effects of HT and TY are shown as percent inhibition of untreated or H_2_O_2_-stimulated fluorescence and represented as the mean ± SEM of three independent replicates carried out in triplicate. ^†^ MCF7; ^‡^ MDA-MB-231; * MCF10A indicates significant differences.

Intracellular reactive oxygen species (ROS) were quantified by the dichlorofluorescein diacetate (DCFH-DA) assay using a microplate reader. Results showed a dose-dependent decrease in ROS level of MCF10A cells treated for 24 h with either HT or TY. However, HT and TY failed to significantly decrease intracellular ROS level in either MCF7 or MDA-MB-231 cells (Figure 2(a)). While HT reduced ROS level by up to 20% in MCF7 cells, this reduction was not considered statistically significant (*p* = 0.34).

H_2_O_2_ effectively induced oxidative stress in both, human breast cancer cells and human breast epithelial cells (Figure 2(b)). In order to investigate the *in vitro* preventive effect of HT or TY against H_2_O_2_-mediated oxidative stress, we measured the intracellular ROS level in cells treated with HT or TY for 24 h. As can be seen in Figure 2(c), MCF10A cells treated with HT or TY showed a significant dose-dependent decrease in ROS production compared to the control. In addition, HT was also able to decrease the ROS level in MCF7 and MDA-MB-231 cells induced by H_2_O_2_ exposure. It is worth mentioning that the decrease in ROS level was greater in the breast epithelial cell line than in the breast cancer cell lines. On the other hand, TY did not decrease ROS level in MCF7 or MDA-MB-231 cells at concentrations up to 5000 μM (Figure 2(c)).

IC_20_ and IC_50_ values were defined as the values for 20% and 50% antioxidant inhibition of basal or H_2_O_2_-stimulated fluorescence in DCFH*-*DA probes. The Relative Antioxidant Value (RAV) ratio was found to be a good parameter for the determination of oxidative inhibition profiles. 

RAV= [(IC_20 (PH)_/IC_20 (TOC)_) + (IC_50 (PH)_/IC_50 (TOC)_)]/2

where PH is the compound (simple phenol) and TOC is the reference (α-tocopherol).

Our results showed that TY has a RAV about 46-fold higher than TOC for MCF10A cells whereas HT only has 1.44-fold higher. This indicated much high antioxidant activity of HT compared with TY in normal breast cells, but less than TOC (Table 2). In MCF7 and MDA-MB-231 cells, a 50% antioxidant inhibition was not observed; therefore, RAV ratios were not determined in these cell lines.

Interestingly, in H_2_O_2_-stimulated MCF10A cells, the RAV ratio of TY was 42-fold higher than TOC, indicating a very low antioxidant capacity in normal breast cells. The 0.67-fold difference between RAV ratio of HT and TOC is of particular interest, due to the high antioxidant activity of HT in H_2_O_2_-stimulated MCF10A cells (Table 2).

**Table 2:** Oxidative inhibition in MCF10A cells. IC_20_ and IC_50_ values defined as the values for antioxidant inhibition of basal or H_2_O_2_-stimulated fluorescence in DCFH-DAassays and the Relative Antioxidant Value (RAV) as a parameter for the relative determination of oxidative inhibition profiles compared to α-tocopherol.

	Basal			H_2_O_2_-stimulated		
	HT (µM)	TY (µM)	TOC (µM)	HT (µM)	TY (µM)	TOC (µM)
**IC_20_**	3.52	4.04	4.33	2.66	65.36	2.49
**IC_50_**	65.64	2942.60	31.89	20.66	4244.40	73.50
**RAV**	1.44	46.60	1.00	0.67	42.00	1.00

### 3.5. Effect of HT and TY on Oxidative DNA Damage

**Figure 3:**
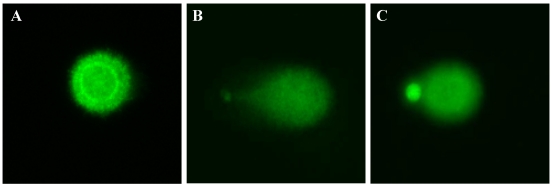
Representative images of Comet assay analysis of MCF10A cells. (**a**) Untreated cell, showing a circular shape indicating absence of DNA damage; (**b**) 10 min. H_2_O_2_ exposed cell, exhibiting a long and bright tail related to DNA strand breaks, indicating DNA oxidative damage; (**c**) 10 min. H_2_O_2_ exposed cell after 24 h of 100 µM HT pretreatment, illustrating the reduction of tail length and fluorescent intensity indicative of reduced DNA damage.

The ability of H_2_O_2_ to induce DNA strand breaks in these human breast epithelial cell lines was examined using the Comet assay. In untreated cells, DNA does not migrate far from the origin when examined by alkaline microgel electrophoresis (Figure 3(a)). Following H_2_O_2_ exposure, control and pretreated breast cells with damaged DNA have the shape of a comet, the tail length and fluorescent intensity of which are related to the number of DNA strand breaks induced by the DNA-damaging agent (Figure 3(b,c)).

**Figure 4:**
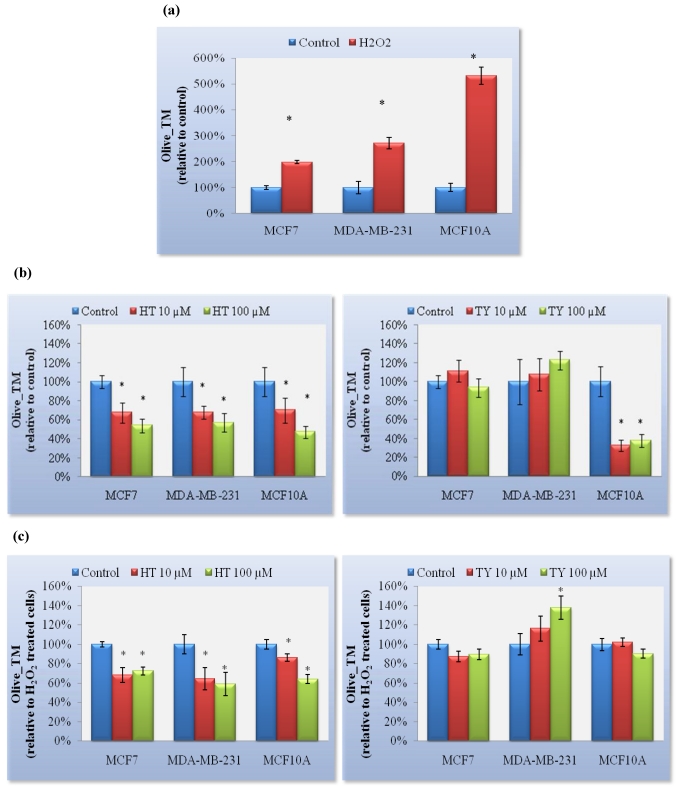
Olive Tail Moment (Olive_TM) as the mean ± SEM for three independent assays. (**a**) After an H_2_O_2_ injury; (**b**) after 24 h of HT or TY treatment, and (**c**) after 24 h of HT or TY treatment followed by an H_2_O_2_ injury.

Breast cells exposed to H_2_O_2_ were effectively DNA damaged and the mean olive tail moment (Olive_TM) was determined by the Comet assay. Breast epithelial cells were the most sensitive to the H_2_O_2_-induced DNA damage (Figure 4(a)).

In unexposed cells, HT reduced DNA damage significantly in MCF7, MDA-MB-231 and MCF10A cells whereas TY only reduced it in MCF10A cells (Figure 4(b)). In H_2_O_2_-exposed cells, HT showed a preventive DNA damage effect in the three cell lines whereas TY was unable to reduce Olive_TM in any of the cell lines; indeed, in MDA-MB-231 cells, TY increased Olive_TM significantly.

## 4. Discussion

There is some scientific evidence relating Mediterranean dietary pattern with a lower incidence of cardiovascular diseases and cancer, among other diseases. Virgin olive oils (VOOs) represent the main source of fats in this diet and it has been demonstrated that consumption of VOOs reduces human arterial hypertension, lipid peroxidation of membranes, tumor incidence and number of tumors [19,20]. Minor compounds play a key role in VOOs’ healthy properties. Among them, phenols have demonstrated healthy bioactivity properties. Interest in phenolic compounds has increased greatly, with attention being focused on finding naturally occurring antioxidants for foods or medical uses to replace synthetic antioxidants that, in some cases, have been reported to be carcinogenic [21].

HT and TY are two of the major simple phenols present in VOOs as simple form or conjugates [2].Bioavailability studies have demonstrated that they are dose-dependently absorbed in animals and humans after olive oil ingestion [22], accumulated in the body and, finally, systemically exert biological effects [23]. 

The present work describes the antioxidant capacity of HT and TY molecules using chemical and cellular assays and their relationship with proliferation of human breast tumor *vs.* normal cells.

HT and TY are structurally identical except that HT has an extra -OH group forming a catechol group, which is considered responsible for its higher antioxidant activity. This catechol group is able to stabilize free radicals through the formation of intermolecular hydrogen bonds [8]. In our chemical analysis, the catechol phenol HT exhibited a strong antioxidant activity in DPPH, ABTS and ORAC assays, while TY, without a catechol group, showed a weak antioxidant activity in DPPH assay. Remarkably, TY acts as an efficient scavenger against ABTS and AAPH radicals, although to a lesser extent than HT, indicating the minor importance of the cathecol group in cationic or peroxylic radicals’ scavenging activities. These results are in agreement with those previously reported by Visioli *et al*. [8] affirming that HT and, to a lesser degree, TY are more potent scavengers of free radicals than vitamin E.

Although nowadays there is no scientific evidence relating to the physiological concentrations of HT or TY after olive oil ingestion, some authors have suggested it could be between 10 and 100 μM [24]. Cell treatment with HT or TY in the range of their possible physiological concentrations (1-100 μM) did not have any effect on cell proliferation in any of the cell lines studied, independently of the exposure times. However, HT dramatically reduced the viability of MCF7, MDA-MB-231 and MCF10A cell lines when used at concentrations from 200 μM to 1000 μM. Fabiani *et al*. described such an effect in colon adenocarcinoma HT29 cells [25]. Furthermore, HT and TY did not alter the cell cycle or induce apoptosis in these cell lines. Although these results are in agreement with those achieved in LLC-PK1 renal cells, they are in contrast with results in human promyelocytic leukaemia HL60 cells with a noticeable antiproliferative, cell cycle arrest and apoptotic effect of HT. Otherwise, TY showed no antiproliferative effect in HL60 cells [7,25].

HT or TY’s inability to inhibit breast cancer cell proliferation at the assayed times and concentrations, suggests that they cannot protect against breast cancer once developed. Quiles *et al*. [24] described the lack of inhibition of HT or TY in PC3 cells treated with 10 to 250 μM, as did Menendez *et al*. [26] in SKBR3 and MCF7 cells after 5 days of HT or TY treatments in the range of 6.25 to 100 μM. Moreover, Sirianni *et al*. [27] recently described the dose-dependent inhibition of MCF7 cell proliferation by HT and oleuropein (OL) with treatments of 1 to 100 μM; cell growth was induced by 17-β-estradiol (E_2_). In addition, HT and OL are not able to interfere with estrogen action through competition with estrogen receptors (ER), which are responsible for activation of the gene expression involved in cell proliferation.

In order to clarify how nutritional antioxidants are able to prevent or treat oxidative damage, Berger [28] affirmed that nutrients cannot treat an installed disease, such as gastrointestinal cancer, but that they may prevent its promotion. Indeed, the answer to the question: “Can installed damage caused by ROS be treated by antioxidant nutrients?” is “probably not”, but the answer to the question: “Can oxidative damage be treated nutritionally?” is “yes” [28]. 

Growing evidence supports the hypothesis that risk factors such as lifestyle, age, environment, diet, drinking, smoke, *etc.* are determinants in breast neoplastic transformation, and are closely associated with a chronic increase in the basal level of oxidative stress. A decrease in oxidative stress state could prevent the development of tumors and, potentially, cancer. In fact, serum markers for oxidative DNA damage have been shown to increase in women diagnosed with breast cancer [29]. On the other hand, it has been suggested that consumption of VOOs, which are particularly rich in phenolic antioxidants, such as HT and TY, should afford considerable protection against breast cancer by inhibiting oxidative stress [2]. In our study we demonstrated that HT and TY reduce basal and H_2_O_2_-induced ROS level in breast epithelial MCF10A cells, whereas TY failed to reduce both in MCF7 or MDA-MB-231 cells and HT only reduced H_2_O_2_-induced ROS level slightly in breast cancer cells. These results point to a differential antioxidant activity of both compounds between normal breast and tumor cells as we described for squalene [16]. Thus, we suggest that HT and TY could prevent oxidative stress in normal breast cells, thereby preventing the initiation of a chain of reactions to transform normal cells into cancer cells. Noticeably, it is necessary to use a much larger amount of TY to obtain the same ROS reduction level as HT in MCF10A cells (Table 2). Up to 100 μM concentrations of HT and TY used in the present study are probably within the physiological range. However, 500 to 5000 μM of TY exceed this range and could be regarded as being in the pharmacological range.

Di Bendeto *et al.* [11] described differences between HT and TY in inhibiting cell-mediated oxidation of LDL (100% HT *vs.* 40% TY) in J774 A.1 macrophage cells due to its intracellular presence. Thus, time-dependent TY, accumulated inside the cell was effective only at later time-points (24 h) or at higher concentrations than HT, which was rapidly found inside the cells and disappeared within 18 h. Thus, we can presume a quick antioxidant defense by HT followed by a slower defense by TY upon VOO intake.

Estrogens, known human breast pro-carcinogens, exert their actions by two mechanisms; the ER-dependent mechanism, involving the activation of ER and subsequent stimulation of cell growth and proliferation [30] or the ER-independent mechanism, involving the generation of genotoxic estrogen metabolites, which are highly reactive and damage DNA by the formation of free radicals and consequently ROS [30]. In accordance with Sirianni *et al.* [27], HT inhibition of E_2_-induced MCF7 proliferation does not involve the ER-dependent mechanism but points to an inhibition of the E_2_ signaling pathway. Felty *et al. * [31] identified mitochondria as a major source of E_2_-induced ROS (mtROS) in breast cancer cells and described mtROS as a messenger involved in signaling pathways of cell proliferation control, increasing the transcription of cell cycle genes. These authors found the same amount of mtROS in ER-negative MDA-MB-468 cells and in ER-positive MCF7 or T47D cells, suggesting that mtROS production does not depend on the presence of ER in breast cancer cells. If mtROS acts as a messenger in breast cancer proliferation, it could explain why an antioxidant such as HT reduces E_2_-induced cell proliferation, as described by Sirianni *et al.*, whereas in the same concentrations without E_2_ stimulation we do not detect any significant growth alteration.

Cellular protection against oxidative stress is provided by two types of antioxidants; direct antioxidants with a redox activity; and indirect antioxidants (redox active or not) which activated the Nrf2/ARE pathway resulting in transcription of phase II enzymes such as glutathione S-transferase, NAD(P)H: quinone oxidoreductase 1 or glutathione reductase [32]. In addition to the fact that HT and TY act as direct antioxidants, they could also be indirect antioxidants activating the nuclear factor-like 2 (Nrf2). Nrf2, considered a key factor in the cellular defense mechanisms against oxidative stress, might be induced more strongly in MCF10A cells than in MCF7 and might have little or no effect in MDA-MB-231 cells, explaining the differential protection effect of HT and TY on intracellular ROS level. To the best of our knowledge, until now, only Liu *et al*. [33] have described the protection of HT on ARPE-19 human retinal pigment epithelial cell line from oxidative stress induced by acrolein, a major component of cigarette smoke. Further studies will be necessary to elucidate the possible Nrf2/ARE pathway intervention of HT and TY differential antioxidant activity in breast cell lines.

HT has been described as preventing DNA damage beyond its antioxidant capacity, as it can affect a range of enzymes, including cyclooxygenase and NAD(P)H oxidase while TY has no protective effect [8]. In accordance with these authors, our findings point to a protective effect of HT against basal and H_2_O_2_-induced DNA damage regardless of the breast cellular type, whereas TY only has a protective effect on ROS basal level in non-tumoral breast cells.

Both compounds reduce intracellular ROS level and oxidative DNA damage in normal breast cells. This could protect against cellular mutations, preventing carcinogenesis. However, when the disease has occurred, oxidative status in the malignancy place is altered. In this condition, while HT still protects non-tumor breast cells against DNA damage, TY fails to protect them at physiological concentrations. Although HT contributes to reduce DNA damage in normal breast cells, it protects breast tumor cells too. Accordingly, our results must be interpreted carefully, because a reduction of DNA damage in cancer cells might promote cell growth and might inhibit the action of anthracycline chemotherapeutic agents, such as doxorubicin, which induces apoptosis of cancer cells by the oxidative damage resulting from enhanced oxidative state of the cells or, in contrast, might reduce ROS messenger signaling of proliferation resulting in a reduction of tumor cell growth. In any case, we have not detected any modulation of the growth activity *in vitro* in breast cancer cells after HT or TY treatment at the assayed times. 

In this paper, HT has been described as an antioxidant compound with higher activity than TY and related to the prevention of breast cancer, but we must not forget that VOO´s minor compounds can interact with each other, potentiating or inhibiting the effects described for each component alone. Our results indicate some healthy properties of these two simple phenols which may be of interest in pharmacology or as a nutritional supplement or could even lead to establishing the ideal concentrations of each component in VOOs in order to label it as a healthy oil. However, we must be prudent about extrapolating these results regarding epidemiological olive oil health impacts. Future work is needed to investigate these synergetic or inhibitory effects.

## 5. Conclusions

The simple phenol HT could contribute to the preventive cancer activity attributed to VOOs due to the reduction of oxidative stress and oxidative DNA protection in normal breast cells at physiological concentrations, whereas TY is needed at pharmacological concentrations to reduce oxidative stress and fails to protect DNA damage against an oxidative burst.

Both phenols exert a selective antioxidant defense, preventing oxidation in normal breast cells but not in breast cancer cells, which could be helpful to cancer therapies that increase oxidative stress. HT also prevents induced DNA damage in cancer cells, so it might interfere with these therapies.

Although *in vitro* studies have pointed to a preventive role of HT against human breast cancer, the precise mechanisms of action remain to be clarified. Further studies are necessary to elucidate the cellular signaling events that HT and TY target in oxidativestress protection and subsequent breast cancer prevention.
